# Postmenopausal Symptoms and Their Correlates among Saudi Women Attending Different Primary Health Centers

**DOI:** 10.3390/ijerph18136831

**Published:** 2021-06-25

**Authors:** Doaa M. Abdel-Salam, Rehab A. Mohamed, Rawan R. Alruwaili, Farah S. Alhablani, Raghad M. Aldaghmi, Raghad E. ALghassab

**Affiliations:** 1 Family and Community Medicine Department, College of Medicine, Jouf University, Sakaka 72388, Aljouf, Saudi Arabia; 2 Public Health and Community Medicine Department, Faculty of Medicine, Assiut University, Assiut 71526, Egypt; 3 Family Medicine Department, Faculty of Medicine, Suez Canal University, Ismailia 41522, Egypt; rehabali11179@gmail.com; 4 College of Medicine, Jouf University, Sakaka 72388, Aljouf, Saudi Arabia; RawanAlruwaili96@gmail.com (R.R.A.); FarahAlhablani20@gmail.com (F.S.A.); Raghadaldughmi@gmail.com (R.M.A.); raghadksa890@gmail.com (R.E.A.)

**Keywords:** postmenopausal symptoms, MRS, associated factors, reproductive health, psychological wellbeing, Saudi women

## Abstract

(1) Background and objectives: Due to increasing life expectancy, more than one-third of women’s life can be spent in the postmenopausal period. In this period, women have different somatic, psychological, and urogenital symptoms. The present study was done to evaluate postmenopausal symptoms and their correlations among Saudi women attending different primary health centers. (2) Methods: adopting a cross-sectional study was done among 845 postmenopausal women attending different primary health centers. The menopause rating scale (MRS) was utilized to investigate the prevalence and severity of different menopausal manifestations. Data collection was done using a structured anonymous questionnaire disseminated during face-to-face interviews. Analysis of data was done utilizing the SPSS program, version 24. (3) Results: The total MRS score was 15.68 ± 6.85. The mean score of the subscales were: 5.56 ± 2.78 for the somatic domain, 6.04 ± 2.89 for the psychological domain, and 4.08 ± 2.32 for the urogenital domain. Joint and muscle pain (25.2%) and sleep problems (18.6%) were the most prevalent severe/very severe somatic symptoms. The most severe/very severe symptoms of the psychological domain were mental and physical exhaustion (20.2%) and depressed mode (19.2%), while that of the urogenital domain were sexual problems (20.1%) and bladder problems (16.3%). Significant predictors of the subscales of MRS were sociodemographic characteristics such as age, residence, marital status, educational level, and occupation. Furthermore, history of chronic diseases, parity, and menopause duration were significant predictors of the subscales of MRS. (4) Conclusion: women in the present study experience different postmenopausal symptoms. Health care providers should consider this crucial stage of women’s life to help elderly women manage these different postmenopausal manifestations.

## 1. Introduction

Permanent stoppage of menstruation is the definition of menopause [1]. Menopause usually occurs between the ages of 47 and 55 years of age as a gradual process [2]. The absence of menstruation for twelve successive months, excluding other physiological or pathological reasons, can be an indication of menopause [1,2]. Menopause is recognized by many psychological and physiological changes, including somatic manifestations (heart discomfort, hot flushes, and sleeping problems), psychological manifestations (depression, irritability, and anxiety), and urogenital manifestations (sexual problems and urinary incontinence) [3].

Menopausal symptoms were significantly higher among postmenopausal women compared to premenopausal women, which significantly influence the quality of life of postmenopausal women [4]. Socio-demographic variables, psychosocial, cultural, and lifestyle factors can influence the prevalence of postmenopausal symptoms. The predominance of menopausal symptoms varies greatly among women in different regions of the world [5,6].

The most frequently encountered postmenopausal manifestations in Latin America were hot flushes (68.9%) followed by sleep disturbances (68.4%) [7]. The postmenopausal manifestations in Australia were hot flushes and night sweats [8]. In a Nigerian study, the most frequently reported manifestations were muscle and joint problems (59%) [9]. The most prevalent menopausal manifestations were joint pain (90.3%), sleep problems (84.0%), and physical and mental exhaustion (80.0%) in an Egyptian study [10]. The most frequent menopausal symptoms among Saudi women were muscle and joint problems (83.9%), physical and mental exhaustion (80.2%), heart discomfort (73.1%), sleeping disorders (71.2%), hot flashes (71.0%), and irritability (71.0%) [11].

Assessing the severity of menopausal manifestations is done using the menopause rating scale (MRS), which is a well-acknowledged instrument. MRS is a reliable scale for long-term surveillance of menopausal manifestations. The practical application of MRS is encouraged by its high reliability and ease of completion [12].

There is deficient information about assessing menopausal symptoms among Saudi women and no study had been conducted in the Aljouf region in Saudi Arabia. The objectives of the present study, therefore, were to determine postmenopausal symptoms and their correlates among Saudi women in the Aljouf region, Saudi Arabia. The value of this study is that it provides an addition to the literature regarding the postmenopausal symptoms and their correlates among Saudi women. The Saudi community until a recent time has been considered a closed community, especially regarding women-related health issues. Moreover, their Islamic background adds more emphasis on their private issues to be discussed with strangers. Little was known about the actual reproductive health needs of the Saudi women, and the willingness to participate in the studies related to their reproductive issue and exploring their privacy was limited. Nowadays, there is a new era of improvement in women’s rights based on 2030 Saudi vision, and this study is a part of exploring the reproductive health of Saudi women and providing a snapshot about the experienced postmenopausal symptoms and their correlations, and consequently providing the study results to the policymakers to enhance the quality of life among postmenopausal women. This study is designed to investigate the hypothesis that Saudi women will experience different postmenopausal symptoms of various degrees and numerous sociodemographic and menopausal correlates influence these symptoms. 

## 2. Participants and Methodology

### 2.1. Study Design and Setting

The current study was cross-sectionally done to evaluate postmenopausal symptoms and their correlates among women attending different primary health centers in the Aljouf region, Saudi Arabia. The Aljouf region exists in the northern part of Saudi Arabia. Data collection was done during the period from January to July 2020. The sampling frame was a list of primary health centers in the Aljouf region, which was taken from the directorate of health affairs.

### 2.2. Sample Size Estimation

The Epi-Info version 7 StatCalc, which is accessible from the Centers for Disease Control (CDC) and the WHO, was utilized for sample size calculation. The authors determined the following criteria after reviewing the available literature to compute the least sample size: population size of 999,999, expected frequency of 50%, confidence level of 95%, and a margin of error of 5%. This gave a sample size of 768 women. The sample was raised to 845 participants after adding 10% as a non-response rate. 

### 2.3. Sampling Technique

**Figure 1:**
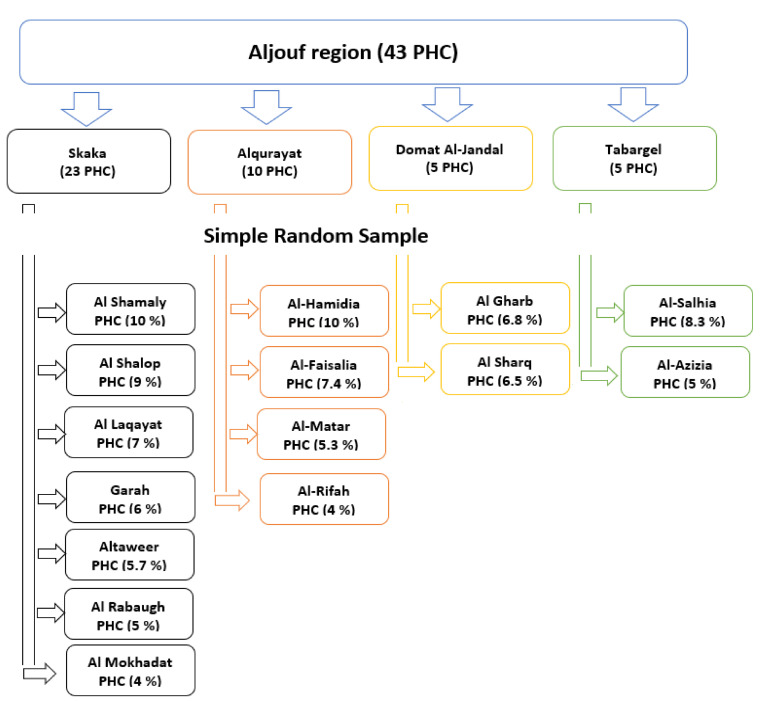
Schematic diagram of the sampling technique.

The Aljouf region in Saudi Arabia has four governorates: Skaka, Alqurayat, Domat Al-Jandal, and Tabargel. There were 43 PHC (primary health center) in the Aljouf region, and 15 centers were selected by simple random sampling technique out of the four governorates. The number of women selected in each primary health center corresponded to the number of women served by this center (Figure 1). All women in the selected primary health centers were invited to voluntarily participate in the present study. The questionnaires were disseminated by the researchers to all women of the selected centers, except those who refused to engage in the study. The inclusion criteria for the enrollment in the study were postmenopausal women with permanent stoppage of menstruation for a year from the last menstrual cycle that is not correlated with a pathological reason. The exclusion criteria in the present study were women using hormonal replacement therapy or those with surgically or medically induced menopause. 

### 2.4. Data Collection Tool

The present study was done using an anonymous structured questionnaire. The questionnaire had 4 sections: socio-demographic variables (age, residence, marital status, level of education, occupation, monthly income in Saudi Riyal (RS), and parity); menstrual history (menopause duration and age at menopause); lifestyle factors (physical activity); and the MRS. Physical activity was characterized as any activity, such as domestic activities or walking, for 20–30 min. Physical activity was classified into 3 levels: infrequent (less than 3 times/week), average (3–5 times/week), and more frequent (more than 5 times/week). The MRS consists of 11 manifestations in 3 domains: somatic domain (hot flushes, sleep problems, heart discomfort, joint and muscle pain); psychological domain (anxiety, depressed mood, irritability, physical and mental exhaustion); urogenital domain (sexual problems, vaginal dryness, and bladder problems) [13]. Each manifestation was scored from none (0) to very severe (4), yielding a score range from 0–44 [13]. According to this score, the severity of postmenopausal symptoms was categorized as: none/little = 0–4, mild = 5–8, moderate = 9–16, severe/very severe = 17 or more [13]. The present study used an Arabic version of the MRS, validated in a previous Egyptian study [10]. The clarity of the questionnaire was tested by conducting a pilot study among 30 women. Data from the pilot study was excluded from the present study. The authors conducted face-to-face interviews with the participants. The participants were selected from the waiting areas of the primary health centers and informed of the goals of the study when eligible.

### 2.5. Statistical Analysis

Data was analyzed using the SPSS program, version 24 (SPSS Inc., Chicago, IL, USA). Number and percentage were used for displaying qualitative data. Mean and standard deviation (SD) were used for presenting quantitative data. ANOVA test was used for comparison between multiple quantitative continuous groups. An independent sample t-test was used for comparison between two quantitative continuous groups. The significant predictors of the subscales of the MRS were determined using regression analysis. A *p*-value ≤ of 0.05 was statistically significant.

### 2.6. Ethical Considerations

The proposal was submitted to the Ethical Review Committee of Jouf University, and data collection was begun after ethical clearance (Approval No: 16-03/41). All procedures were done according to the international guidelines of research ethics and the declaration of Helsinki. The researchers ensured the ethical aspects in the present study by describing the goals of the present study to the participants before filling the questionnaire, acquiring informed written consent from the women who were interested to participate in the study, and guaranteeing the confidentiality of the collected data.

## 3. Results

**Figure 2:**
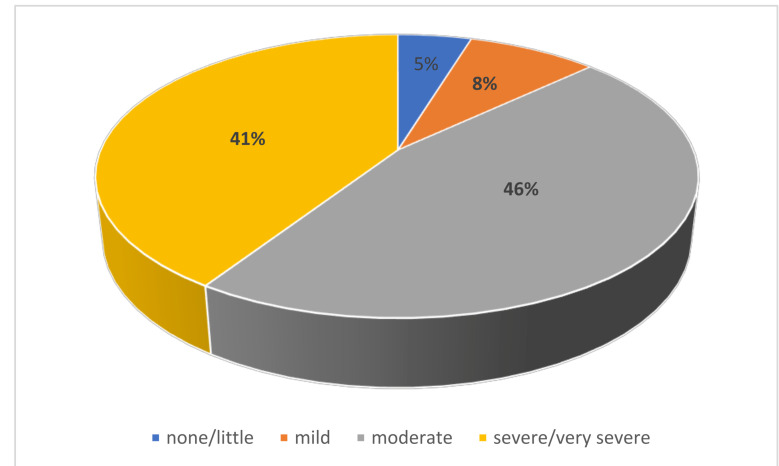
Severity of postmenopausal symptoms according to MRS among Saudi women attending different primary health centers.

The present study included 845 women aged 47–82 years with a mean age ± SD of 54.75 ± 6.76. Table 1 demonstrates the sociodemographic characteristics and menopausal history of the participants. Most of the women in this study were married (77.4%), physically inactive (80.3%), and parous (74.7%). Furthermore, 88.6% of the respondents had an urban residence, and 47.1% were housewives. Regarding the menopausal history, the age of menopause was < 50 years among 76.8% of the participants, and menopause duration was less than 10 years among 87.9%. Table 2 depicts the degree of severity of postmenopausal symptoms among the participants. The most prevalent severe/very severe somatic manifestations were muscle and joint pain (25.2%) and sleep problems (18.6%). Concerning psychological symptoms, the participant complained of mental and physical exhaustion (20.2%) and depressed mode (19.2%) as severe/very severe symptoms. The most severe/very severe symptoms of the urogenital domain were sexual problems (20.1%) and bladder problems (16.3%). The total MRS score was 15.68 ± 6.85. The mean score of the subscales were: 5.56 ± 2.78 for the somatic domain, 6.04 ± 2.89 for the psychological domain, and 4.08 ± 2.32 for the urogenital domain. Regarding the severity of postmenopausal symptoms according to MRS, 46% of the respondents had a moderate degree, 41% had severe/very severe degree, whereas 8% had a mid-degree (Figure 2).

Table 3 shows the sociodemographic and menopausal correlates of the MRS score. MRS score is significantly higher among lower age (*p* = 0.000), rural residents (*p* = 0.005), divorced/widowed (*p* = 0.000), less educated (*p* = 0.000), housewives (*p* = 0.001), and physically inactive (*p* = 0.000). Furthermore, the MRS score is significantly higher among women with low monthly income (*p* = 0.002) and women with a history of chronic diseases (*p* = 0.000). Concerning menopausal correlates, the MRS score is significantly higher among women with menopause duration < 10 years (*p* = 0.000). Significant predictors of the somatic domain in the MRS were age, residence, marital status, parity, menopause duration, and history of chronic diseases (Table 4). Age, residence, marital status, occupation, parity, menopause duration, and history of chronic diseases made a significant contribution to predicting the psychosocial domain in the MRS (Table 5). Table 6 reveals that the significant predictors of the urogenital scale were age, educational level, parity, menopause duration, and history of chronic diseases.

## 4. Discussion

The present study aimed to investigate postmenopausal symptoms and their correlates among Saudi women attending different primary health centers in the Aljouf region, Saudi Arabia. Menopause is a crucial time that not only denotes the end of reproductive capacity in a woman’s life, but is connected to numerous psychological, vasomotor, physical, and sexual problems [14]. The questionnaires in the current study were disseminated during face-to-face interviews instead of being self-completed as some participants had limited educational level. The present study revealed that Saudi women experienced different postmenopausal symptoms with various sociodemographic and menopausal correlates influenced these symptoms. 

The present study findings revealed that the total MRS score was 15.68 ± 6.85, which is more severe than the reported results in a study conducted in Nigeria, where the total MRS score was 14.02 ± 0.44 [15]. Another study conducted in Egypt revealed that the MRS score was 18.4 ± 7.1 in postmenopausal women [16]. However, the current results were consistent with the findings of a study conducted in Abha, Saudi Arabia where the MRS mean score was 15.25 ± 6.01 [17], and with another study in India [18]. These differences can result from the variation of individual responses to menopause and estrogen deficiency due to genetics, lifestyle, socioeconomics, and education.

In the current study, the severity of the psychological symptoms was the highest among the three studied domains. However, in other studies in different countries, the somatic symptoms were the highest [15,19,20,21]. This difference can be explained by the different ways of perception of these symptoms between different countries in addition to the use of different types of scoring. In addition, since urogenital and sexual problems in this culture are not freely addressed, they may be converted into physical and psychological symptoms. They may even assume that those signs are a normal part of aging. 

Among the somatic manifestations shown to be severe by most of the participants were muscle and joint pain followed by sleep problems. These highly reported symptoms are similar to those reported in other studies conducted in Saudi Arabia [11,17]. However, these symptoms are multifactorial, and the women in Saudi Arabia usually suffer from a lack of exercise and Vit D deficiency [22].

Regarding the psychological symptoms reported in this study, the most severe symptoms were depressed mood along with mental and physical exhaustion, which can be explained by the discomfort of the symptoms of menopause and fluctuating hormone levels during this period where women may have bouts of depression and sadness. Moreover, mood disorders are usually prevalent among women, such as anxiety, depression and mental illness, and other psychological problems [23]. As hormones decrease in this period of life, especially estrogen, the main neurotransmitters such as serotonin, dopamine, and endorphins are reduced [24]. Another study in the Mediterranean region revealed that in the psychosocial, anxiety, nervousness and memory loss were the most severe symptoms [19]. 

In the domain of urogenital symptoms, a study conducted in Bahrain reported that the sexual domain has been seriously affected in terms of changes in sexual desire [25]. This is in line with the results of this study, where the most severely reported symptoms of the urogenital domain were sexual problems followed by bladder problems. A study conducted in India reported the same findings where postmenopausal women had advanced symptoms of decreasing sexual desire, avoiding intimacy, and feeling nervous or anxious [20]. Another study revealed that women are vulnerable to diminished sexual desire, depression, back problems, and memory problems, and they eventually suffer declining health and reduced quality of life [26]. In addition, voiding difficulty became worse as the women went through menopause. The deficiency of estrogen hormone during menopause can worsen bladder contraction and cause urethral dysfunction by influencing the urethra’s circulation, which reduces pressure and leads to incontinence [27]. 

Practicing physical activity improves the quality of life of postmenopausal women, and the literature indicated that physical activity is correlated with a drop in hot flushes [28]. One study suggested that the value of physical activity increases during the menopausal transition and supports the theory that menopause can be a real opportunity, as it may cause lifestyle change [29]. In the current study, postmenopausal symptoms increase among women with reduced physical activity (less than 3 times/week). This is consistent with Al-Musa et al.’s study, which reported that practicing exercise more than five times/week was significantly correlated with a lower MRS score [17]. Moreover, exercise increases endorphins in the blood, which help to decrease vasomotor manifestations and has advantageous effects on the mood, well-being, sleeping disorder, and cognitive functions of women [30]. 

Among other factors that influence post-menopausal life are some of the demographic characteristics of post-menopausal women, such as marital status, educational level, and social and economic level [23]. Increased MRS mean score was prevalent among less-educated women and those belonging to the lower socioeconomic level, which is in line with the results of a study done in the Mediterranean region [19]. More educated women have better income, more opportunities, have better access to health services, and benefit better from medical advice. In addition, women with poor socio-economic conditions are predisposed to depression and other psychological issues [31]. 

A study conducted in a rural area of Egypt revealed that married women had a lower MRS score than unmarried, divorced, separated, and widowed women, which is consistent with this study. The author suggests that married women may have higher income and social support because they have a regular sexual life and have families for support. Furthermore, women with good family relations and positive partner support will experience less severe menopausal symptoms [32].

The status of chronic diseases was also found to be significantly associated with a greater MRS score in the current study. This can be demonstrated by the exaggerating effects of the chronic disease itself, psychological effects because of chronic disease, and the aging process that may all affect the occurrence of menopausal manifestations. 

The current study revealed a significant negative correlation between MRS score and duration of menopause. The results of other research confirm the current study findings; that women in late menopause are less prone to the severity of symptoms associated with menopause [33,34]. 

Early onset of menopause usually occurs in nulliparous women because of the anovulatory period of parous women that leads to postponement of menopause [35,36]. The present study revealed that the MRS score is significantly higher in nulliparous women. Humeniuk et al. revealed that residing in a rural area has a contributing effect for enhancing the risk of postmenopausal symptoms, which was established in the present study [37]. Employment ameliorates the health indicators and is associated with a low occurrence of menopausal symptoms [38]. Employment was significantly associated with MRS in the present study, as working women had a lower MRS score than housewives. Old women have fewer postmenopausal symptoms compared to young women [34]. This is confirmed in this study as the MRS score decreased as age increased.

There are some limitations involving the present study. This cross-sectional study did not exclude the confounding effect of the natural aging process that may affect the experienced postmenopausal manifestations. Furthermore, the MRS depends on recall data, so recall bias can be expected in this study.

## 5. Conclusions and Recommendation

Postmenopausal women in the present study complain of various somatic, psychological, and urogenital symptoms. Age, residence, occupation, marital status, level of education, and history of chronic diseases were significant predictors of the subscales of MRS. In addition, parity and menopause duration were significant predictors of the subscales of MRS. Addressing postmenopausal symptoms among elderly women is of crucial importance, and health care providers must be careful about this important stage in women’s life to help to alleviate postmenopausal symptoms.

## Figures and Tables

**Table 1:** Sociodemographic characteristics and menopausal history of Saudi women attending different primary health centers.

	No. (*n* = 845) (%)
**Age**	
47–57	628 (74.3%)
58–68	168 (19.9%)
69–79	42 (5.0%)
≥ 80	7 (0.8%)
Mean ± SD (Range)	54.75 ± 6.76 (47–82)
**Residence**	
Urban	749 (88.6%)
Rural	96 (11.4%)
**Marital status**	
Married	654 (77.4%)
Divorced/widowed	191 (22.6%)
**Level of Education**	
Illiterate or read and write	107 (12.7%)
Primary or preparatory or secondary	527 (62.3%)
University education or above	211 (25.0%)
**Occupation**	
Worker	223 (26.4%)
Housewife	398 (47.1%)
Retired	224 (26.5%)
**Monthly income**	
<5000 RS	238 (28.2%)
5000–7000 RS	357 (42.2%)
>7000 RS	250 (29.6%)
**Physical activity (times/week)**	
<3 times	678 (80.3%)
3–5 times	149 (17.6%)
>5 times	18 (2.1%)
**Parity**	
Parous	631 (74.7%)
Nulliparous	214 (25.3%)
**Age at menopause**	
<50 years	649 (76.8%)
≥50 years	196 (23.2%)
**Menopause duration**	
<10 years	743 (87.9%)
≥10 years	102 (12.1%)
**History of chronic diseases**	
Yes	597 (70.7%)
No	248 (29.3%)

RS (Saudi Riyal).

**Table 2:** Prevalence of postmenopausal symptoms among Saudi women attending different primary health centers.

	None	Mild	Moderate	Severe/Very Severe
	No. (%)	No. (%)	No. (%)	No. (%)
**Somatic symptoms**				
Hot flushes	245 (29.0%)	267 (31.6%)	221(26.2%)	112 (13.3%)
Heart discomfort	232 (27.5%)	272 (32.2%)	245 (29.0%)	96 (11.4%)
Sleep problems	196 (23.2%)	217 (25.7%)	275 (32.5%)	157 (18.6%)
Muscles and joint pain	167 (19.8%)	214 (25.3%)	251 (29.7%)	213 (25.2%)
**Psychological symptoms**				
Depressed mode	159 (18.8%)	218 (25.8%)	306 (36.2%)	162 (19.2%)
Irritability	201 (23.8%)	210 (24.9%)	283 (33.5%)	151(17.9%)
Anxiety	194 (23.0%)	228 (27.0%)	265 (31.4%)	158 (18.7%)
Physical and mental exhaustion	153 (18.1%)	228 (27.0%)	293 (34.7%)	171 (20.2%)
**Urogenital symptoms**				
Sexual problems	184 (21.8%)	215 (25.4%)	276 (32.7%)	170 (20.1%)
Bladder problems	294 (34.8%)	171 (20.2%)	242 (28.6%)	138 (16.3%)
Vaginal dryness	220 (26.0%)	269 (31.8%)	232 (27.5%)	124 (14.7%)

**Table 3:** Sociodemographic and menopausal correlates of MRS score among Saudi women attending different primary health centers.

	MRS Score	*p*-Value
	Mean ± SD	
**Age**		0.000 **
47–57	33.42 ± 4.53	
58–68	30.64 ± 3.62	
69–79	15.41 ± 1.78	
≥ 80	14.56 ± 2.45	
**Residence**		0.005 *
Urban	13.85 ± 1.45	
Rural	15.92 ± 2.06	
**Marital status**		0.000 *
Married	14.69 ± 1.71	
Divorced/widowed	19.06 ± 2.01	
**Level of Education**		0.000 **
Illiterate or read and write	16.8037 ± 1.53	
Primary or preparatory or secondary	16.5028 ± 2.11	
University education or above	14.0758 ± 2.45	
**Occupation**		0.001 **
Worker	14.43 ± 1.08	
Housewife	16.52 ± 3.94	
Retired	15.43 ± 2.05	
**Monthly income**		0.002 **
<5000 RS	17.43 ± 3.24	
5000–7000 RS	15.45 ± 2.49	
>7000 RS	14.33 ± 1.63	
**Physical activity (times/week)**		0.000 **
<3 times	16.19±3.14	
3–5 times	14.18 ± 2.76	
>5 times	8.61 ± 1.75	
**Parity**		0.000 *
Parous	14.70 ± 2.55	
Nulliparous	18.55 ± 3.13	
**Age at menopause**		0.344 *
<50 years	15.80 ± 2.92	
≥50 years	15.27 ± 1.59	
**Menopause duration**		0.000 *
<10 years	22.39 ± 4.01	
≥10 years	14.76 ± 2.45	
**History of chronic diseases**		0.000 *
Yes	16.79 ± 3.67	
No	13.01 ± 2.52	

* Independent *t* test; ** ANOVA test; RS (Saudi Riyal).

**Table 4:** Linear regression model showing the predictors of the somatic domain in MRS score among Saudi women attending different primary health centers.

Variables	B	*t*	*p*-Value	95% Confidence Interval	
				Lower Limit	Upper Limit
Age	−0.198	−5.209	0.000	0.051	0.112
Residence (rural)	0.079	2.558	0.011	0.161	1.218
Marital status (divorced/widowed)	0.120	3.671	0.000	0.372	1.227
Level of education (less than university)	−0.067	−1.907	0.057	−0.009	0.633
Occupation (housewife)	−0.015	−0.495	0.621	−0.171	0.287
Monthly income (<7000 RS)	−0.056	−1.702	0.089	−0.055	0.768
Physical activity (<3 times/week)	−0.046	−1.288	0.198	−0.424	0.088
Parity (nulliparous)	−0.109	−3.630	0.000	−1.008	−0.300
Age at menopause (<50 years)	−0.051	−1.700	0.090	−0.727	0.052
Menopause duration (<10 years)	−0.173	−4.758	0.000	0.865	2.079
History of chronic diseases (Yes)	0.216	7.026	0.000	−1.689	−0.952

Reference groups: residence (urban), marital status (married), educational level (university or above), occupation (working), monthly income (>7000 RS), physical activity (≥3 times/ week), parity (parous), age at menopause (≥50 years), menopause duration (≥10 years), history of chronic disease (no).

**Table 5:** Linear regression model showing the predictors of the psychological domain in MRS score among Saudi women attending different primary health centers.

Variables	B	*t*	*p*-Value	95% Confidence Interval	
				Lower Limit	Upper Limit
Age	−0.211	−5.214	0.000	0.056	0.124
Residence (rural)	0.078	2.376	0.018	0.123	1.295
Marital status (divorced/widowed)	0.103	2.956	0.003	0.240	1.187
Level of education (less than university)	−0.033	−0.876	0.381	−0.197	0.514
Occupation (housewife)	−0.077	−2.362	0.018	−0.560	−0.052
Monthly income (<7000 RS)	−0.037	−1.054	0.292	−0.211	0.700
Physical activity (<3 times/week)	−0.022	−0.572	0.567	−0.366	0.201
Parity (nulliparous)	−0.088	−2.761	0.006	−0.943	−0.159
Age at menopause (<50 years)	−0.005	−0.147	0.883	−0.464	0.399
Menopause duration (<10 years)	−0.138	−3.564	0.000	0.549	1.894
History of chronic diseases (Yes)	0.095	2.906	0.004	−1.014	−0.196

Reference groups: residence (urban), marital status (married), educational level (university or above), occupation (working), monthly income (>7000 RS), physical activity (≥3 times/ week), parity (parous), age at menopause (≥50 years), menopause duration (≥10 years), history of chronic disease (no).

**Table 6:** Linear regression model showing the predictors of the urogenital domain in MRS score among Saudi women attending different primary health centers.

Variables	B	*t*	*p*-Value	95% Confidence Interval	
				Lower Limit	Upper Limit
Age	−0.288	−7.382	0.000	0.073	0.125
Residence (rural)	0.031	0.977	0.329	−0.228	0.681
Marital status (divorced/widowed)	0.008	0.232	0.817	−0.411	0.324
Level of education (less than university)	−0.085	−2.335	0.020	−0.604	−0.052
Occupation (housewife)	−0.011	−0.342	0.732	−0.231	0.163
Monthly income (<7000 RS)	−0.061	−1.806	0.071	−0.028	0.678
Physical activity (<3 times/week)	−0.002	−0.044	0.965	−0.215	0.225
Parity (nulliparous)	−0.126	−4.097	0.000	−0.938	−0.330
Age at menopause (<50 years)	−0.078	−2.528	0.763	−0.765	−0.096
Menopause duration (<10 years)	−0.078	−2.082	0.038	0.032	1.075
History of chronic diseases (Yes)	0.197	6.230	0.000	−1.323	−0.689

Reference groups: residence (urban), marital status (married), educational level (university or above), occupation (working), monthly income (>7000 RS), physical activity (≥3 times/ week), parity (parous), age at menopause (≥50 years), menopause duration (≥10 years), history of chronic disease (no).

## Data Availability

Data are available from the corresponding author upon reasonable request.
